# Pericardiocentesis Simulation Model: Low Cost, Easy to Assemble, Effective Task Trainer

**DOI:** 10.7759/cureus.14475

**Published:** 2021-04-13

**Authors:** Robert Angert, Orna Rosen

**Affiliations:** 1 Pediatrics and Neonatology, NYU Langone Medical Center, New York, USA; 2 Pediatrics and Neonatology, Children's Hospital at Montefiore, New York, USA

**Keywords:** pericardiocentesis, pericardial effusion, simulation trainer, pediatrics & neonatology, neonatology

## Abstract

Pericardial effusion is a potentially lethal condition. If it is discovered early, it can be treated by pericardiocentesis under controlled condition with imaging by experienced care providers. If it is diagnosed at a later stage with clinical compromise, then an emergent procedure might be necessary. Since it is encountered infrequently, many providers may have little or no experience in managing the condition and performing a life-saving pericardiocentesis.

This is a technical report that describes the creation of a neonatal model for pericardiocentesis. This is a high-fidelity, low-cost model that is simple to create. Materials that are inexpensive and easy to obtain are utilized to make the model. Neonatal care providers, including residents, fellows, nurse practitioners, physician assistants, and attendings, can practice with this model. In some medical centers, an echocardiogram or bedside ultrasound is available to guide needle insertion; however, practicing the procedure on a model provides valuable experience. This model is designed to teach the performance of unguided pericardiocentesis without the use of simultaneous imaging.

Included with this technical report are a supply list, a checklist, and a suggested scenario that can be used in association with this model. In this article, we have discussed our own experience and described lessons learned about training neonatal care providers in pericardiocentesis.

## Introduction

Cardiac tamponade is a catastrophic event for any patient. Newborn babies, both full term or pre-term, can experience cardiac tamponade as a result of an air leak syndrome [1] or as a complication from a central venous catheter with leakage of fluids or infusate (blood and total parenteral nutrition [TPN]) [2] into the pericardial space, among other causes. Cardiac tamponade leads to shock, metabolic acidosis, and death. It is crucial to know when to suspect and how to diagnose and treat a cardiac tamponade as rapid progression and death may occur [3].

The procedure is performed while the manikin is in the supine position. A 5-cc syringe is attached to a three-way stopcock and a 21- or 22-gauge needle or angiocath. If time permits, extension tubing is inserted between the needle and the syringe. The skin is prepared with an appropriate antibacterial agent, and a sterile field is created with sterile drapes. The needle is inserted 0.5-1 cm inferior to the xyphoid, aiming toward the acromion process of the left shoulder with an angle of 30 degrees to 40 degrees from the skin. The needle is advanced while the operator aspirates gently, until air or fluid enters the syringe. If the condition being treated is a pneumopericardium, once the syringe is full of air, the syringe is evacuated by operating the three-way stopcock to allow its release and then repositioning the valve to close the system and more air is removed. This procedure may be repeated until all air is evacuated. If the tamponade is caused by fluid, then the operator continues to aspirate the fluid with the needle or catheter until the flow ceases. The stopcock is closed toward the baby, and the aspirated fluid is given to the assistant to be sent for laboratory analysis. If more fluid is present, another syringe may be attached; the stopcock is now opened toward the baby and the process continues. Once enough air or fluid is drained, there should be a clinical improvement [4].

In our institutions, we practice pericardiocentesis twice yearly: (1) every July in our “Boot Camp” for the neonatal fellows, nurse practitioners, physician assistants, and attending physicians and (2) mid-year when we teach the critical procedures necessary for neonatology care providers to master. In case of a cardiac tamponade development in a newborn, the speed of diagnosis and treatment are crucial for saving the baby’s life, and for this reason we use different clinical scenarios aimed at improving the care providers’ ability to diagnose a tamponade and to effectively drain it.

## Technical report

To create the model of cardiac tamponade, we modified a low-fidelity Laerdal™ neonatal resuscitation manikin (Model #240-00001, Laerdal Medical Corporation, USA) by inserting into the chest a simulated neonatal heart with pericardial tamponade created as described below.

Creation of fluids

To create the simulated blood, we added 0.5 cc of red food coloring to 5 cc of water. The simulated body fluid liquid or TPN is created by adding two to three drops of betadine to 15-20 cc of water.

Filling balloons/gloves to simulate a heart with pericardial effusion

**Figure 1:**
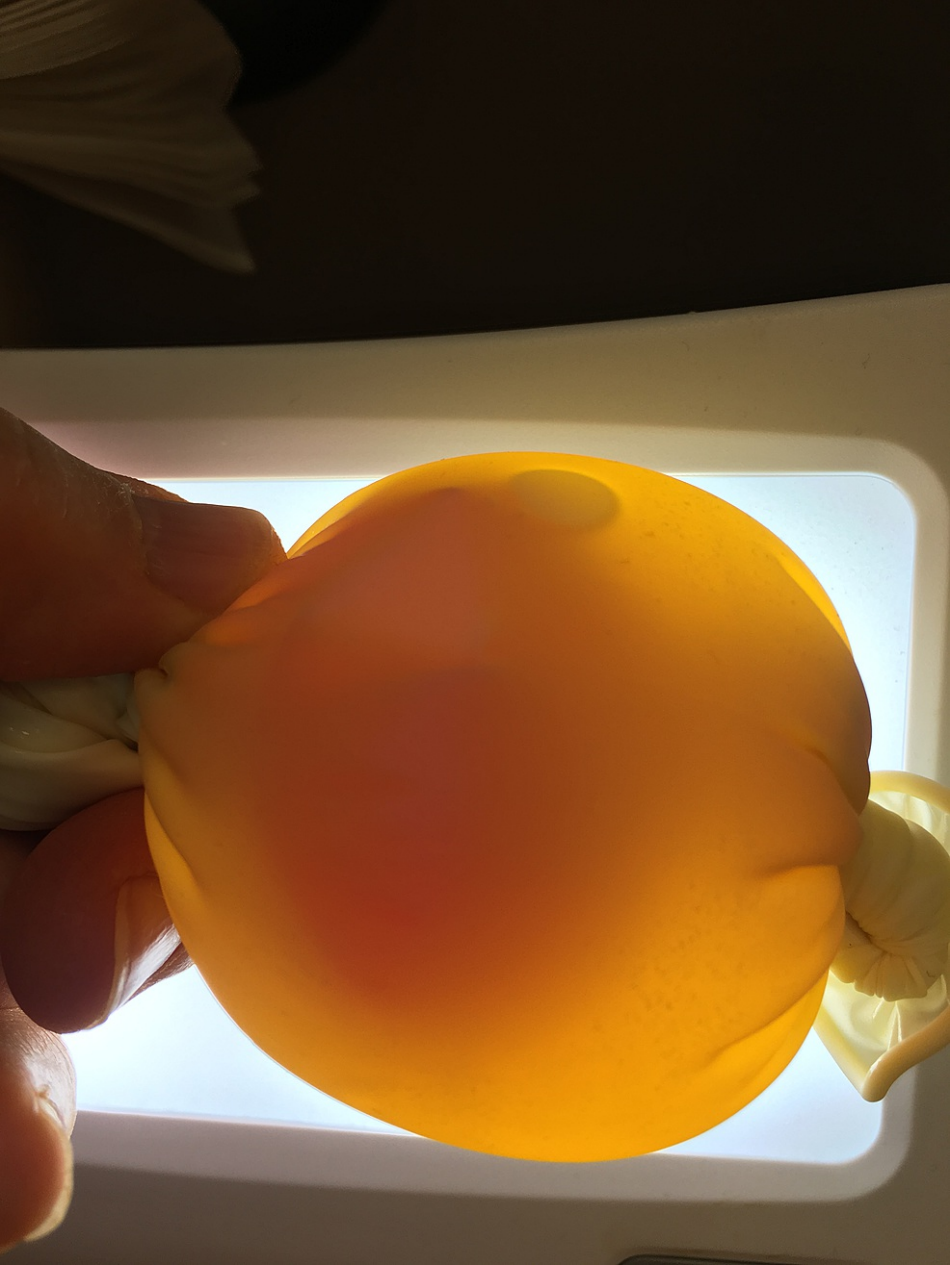
Balloon with red-colored water within a glove containing yellow-colored water

A small water balloon is filled with 5-10 ml of the simulated blood and securely tied off. This balloon is placed into a latex glove with the fingers tied off and cut. The glove is then filled with 15-20 cc of the simulated body fluid or TPN, and the wrist area is tied off, removing as much air as possible (Figure 1).

Inserting the simulated heart with pericardial effusion into the manikin

**Figure 2:**
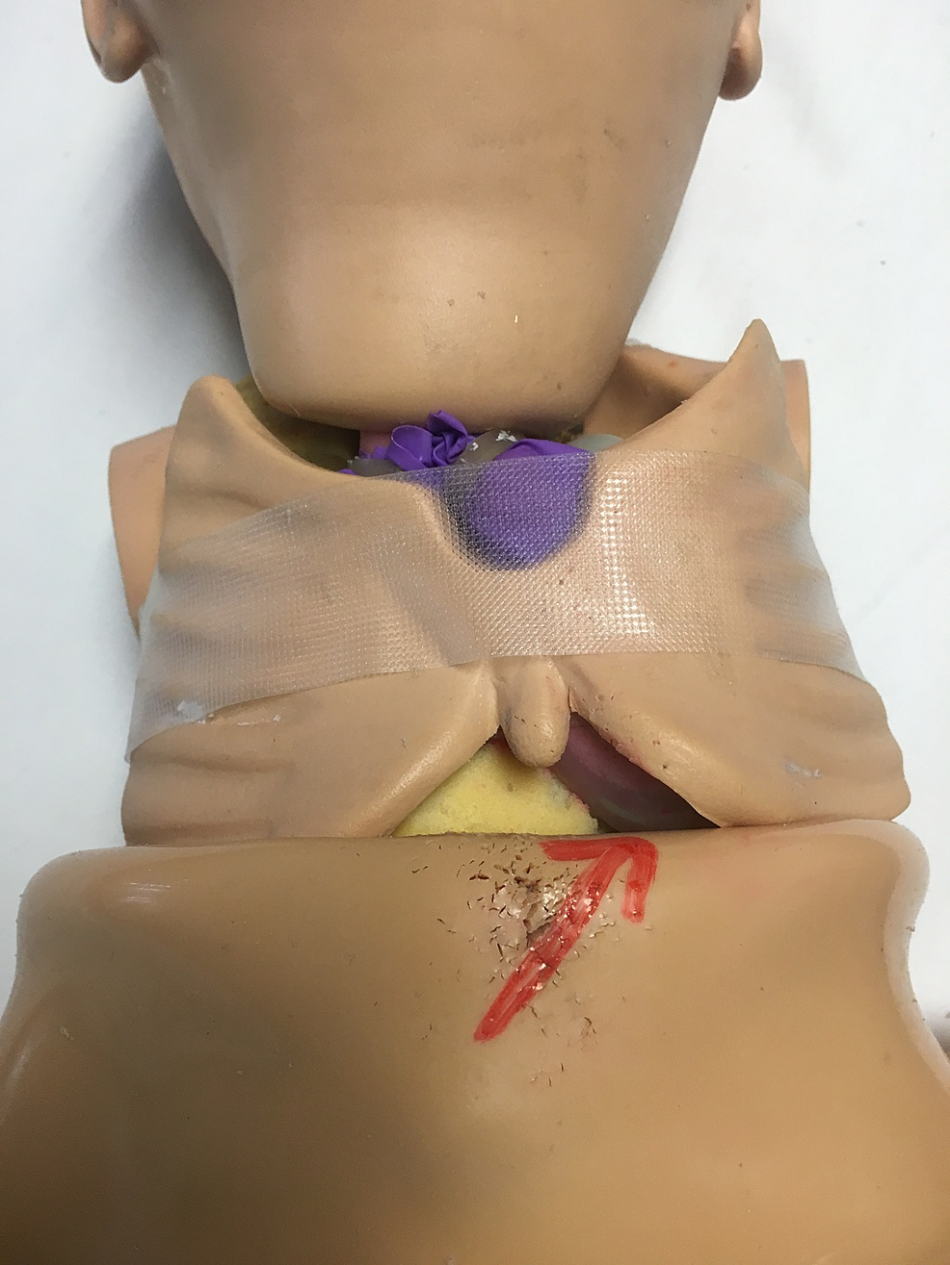
Simulation manikin with plastic pulled back to reveal chest cavity Foam is removed, and the balloons are inserted where the arrow points.

This model has removable skin, and the chest cavity can be accessed after removing the arms that are affixed to the shoulders with a screw-type mechanism. There is a removable plastic rib cage that has a xiphoid process, which will act as a landmark for the procedure. The left chest cavity has a foam filler that must be removed or trimmed to make room for the simulated heart. The simulated heart is secured in the chest cavity with adhesive tape; the removable plastic rib cage is placed on top; the skin is replaced; and the arms are reattached to the manikin. It is important to be able to palpate the xyphoid that is located at the bottom of the rib cage since this is the landmark for needle insertion (Figure 2).

**Figure 3:**
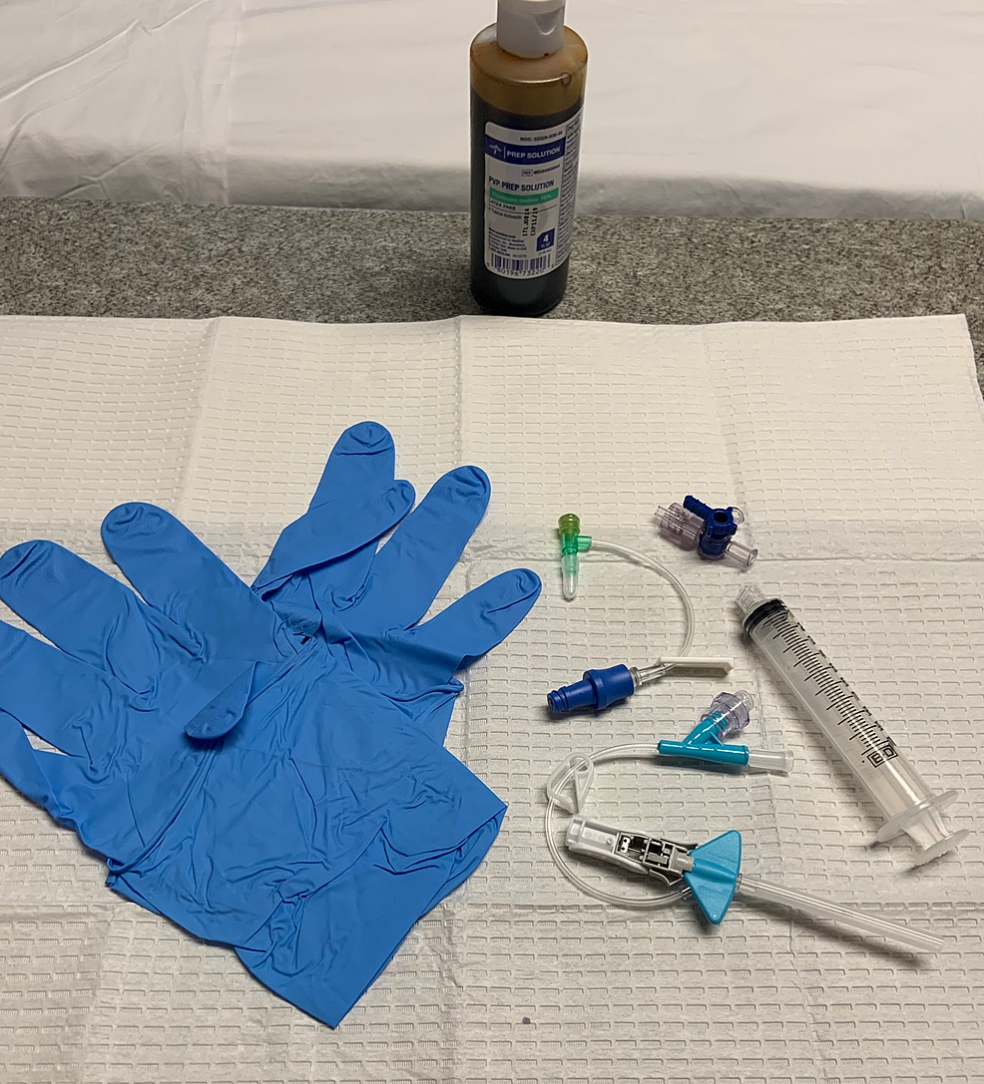
Syringe, three-way stopcock, and angiocatheter with extension tubing

A 10-20 ml syringe is attached to a three-way stopcock and a 22-gauge angiocatheter (Figure 3).

Simulating the procedure

**Figure 4:**
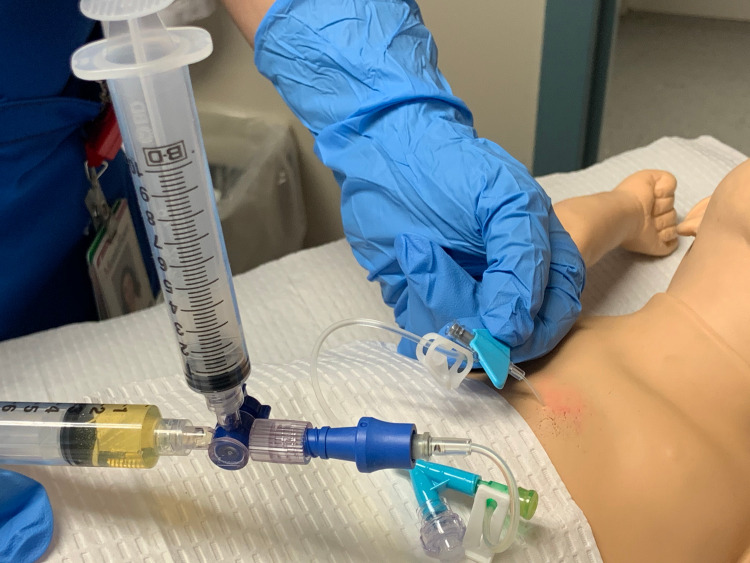
Insertion of needle into model to simulate pericardiocentesis

The learner prepares the area as per unit protocol, inserting the needle 0.5-1 cm inferior to the xyphoid process at an angle of 30 degrees to 40 degrees from the skin, aiming at the left shoulder. The learner gently aspirates as the syringe is advanced until there is yellow fluid, indicating that the needle is in the correct place. If simulated blood is aspirated, it means that the heart was penetrated, and the needle was advanced too far (Figure 4).

Clinical scenario

Learning Objectives

Cognitive: Recognize the clinical presentation of cardiac tamponade due to pericardial effusion.

Understand the importance of sending the recovered fluid for analysis, ordering the indicated laboratory studies.

Technical: Learn how to assemble a needle/angiocath prior in order to penetrate the chest.

Gain proficiency in performing pericardiocentesis through deliberate practice of the procedure in a simulated environment.

Behavioral: Demonstrate teamwork, good communication, and crew resource management during the mock code and procedure.

Case

The patient is a three-day-old infant of a diabetic mother, Ex-40-week gestation male infant with a primary problem of hypoglycemia and who requires no respiratory support. An umbilical venous catheter is in place for high-concentration glucose administration. The baby develops poor perfusion, distress, and initially tachycardia, which evolved within minutes into bradycardia with a heart rate of 50 bmp. The color is reported as gray with cold, poorly perfused extremities. Upon auscultation, there are barely audible heart sounds with a weak pulse present. Neonatal resuscitation is started with the initiation of positive pressure ventilation using a bag and mask.

History

Medical: Status-post dextrose 10% bolus of 2 cc/kg given four times in order to raise the serum glucose.

Baseline lab values: Glucose 55 mg/dL

Oxygen saturations: 75%; HR: 50 bpm

Electrocardiogram: Small QRS complexes consistent with low voltage

Equipment

1. 5 and 10 cc syringes

2. Needles, size 22 or 21 gauge

3. Angiocatheter, size 22

4. Three-way stopcock

5. Extension tubing (T connector)

6. Betadine/sterile gauze

7. Sterile specimen tubes for lab analysis

Manikin Trainer Preparations

Laerdal™ neonatal resuscitation manikin (Model #240-00001, Laerdal Medical Corporation, USA) with pericardiocentesis modification as described in this report.

Additional materials: Printed 12 lead EKG.

Scenario Progression

Expected interventions: Begin standard neonatal code: Give positive pressure ventilation, endotracheal intubation, initiate chest compression, obtain intravenous access, administer fluid bolus, and order chest x-ray.

Likely progression: The patient fails to improve until pericardial effusion is recognized and treated. Oxygen saturations fall to the 50%, heart rate remains 50 bpm due to hypoxemia, and heart sounds are barely audible. Perform bedside ultrasound if available.

Expected endpoint: Drainage of pericardial effusion.

Videotape Guidelines (Priorities to Capture on Videotape)

Video tape the performance of procedure with a priority on capturing communication and teamwork displayed between the participants in the procedure.

Trainee Roles

Two to five code participants: Primary subject inserts needle/IV catheter and secondary subject operates three-way stopcock and removes fluid-filled syringe. 

Additional code participant roles: Manage airway and ventilation, monitor vital signs, and record code events.

Debriefing Points

1. When was the problem recognized? Could it have been recognized sooner?

2. How well did the team function to prepare for and perform the procedure? Are there any opportunities for improvement?

3. Was the recovered fluid the color you expected? If not, what do you think happened? How could this have been prevented?

## Discussion

Cardiac tamponade is a catastrophic event, and if not immediately treated, the patient will progress to cardiac arrest and likely die. In many hospitals, an ultrasound machine is available for bedside use, and it may be used to both diagnose and guide needle placement for pericardial drainage in an emergency. If the procedure is done under the guidance of ultrasound, less risk of complications can be presumed, but at times an unguided tap may be indicated. All neonatal care providers should know the technique of performing a pericardiocentesis, with or without sonographic guidance. We created this model to provide neonatal care providers experience in performing the procedure with the recovery of yellow fluid as a mark of success. We found that the balloon model was easy to affix in the chest cavity, and it effectively simulates the effusion. This reliably leads to successful drainage of the effusion by the trainee. By inserting the inner balloon filled with the simulated blood, we created the simulated heart with the outside balloon simulating the pericardial sac. The gap between the balloons that is filled with yellow fluid to simulate the effusion effectively allows the learners to practice the procedure. If the needle is advanced too far, then the simulated heart is penetrated and blood is seen in the syringe. The teaching point is that the trainee should learn to advance the syringe slowly while applying a constant low suction applied to the syringe plunger once the chest is penetrated and to stop advancing once fluid has been recovered. If the syringe is advanced too far, then the heart will be penetrated. The model is good for two to three sessions prior to replacement of the balloon due to leakage from multiple penetrations.

## Conclusions

Forty-five fellows, providers, and attending physicians have learned through this model. The rare nature of this emergency makes learning in a live situation almost impossible, even in the busiest of intensive care units. By building this simple but effective model, providers can practice this potentially life-saving procedure in simulated environment. Success is determined by direct feedback through draining the pericardiocentesis. If blood is recovered, the learner can easily try again, until the procedure is mastered.

## References

[1] Trujillo MH, Fragachan CF, Tortoledo F (2006). Cardiac tamponade due to pneumopericardium. Cardiology.

[2] Beardsall K, White DK, Pinto EM, Kelsall AW (2003). Pericardial effusion and cardiac tamponade as complications of neonatal long lines: are they really a problem?. Arch Dis Child Fetal Neonatal Ed.

[3] Warren M, Thompson KS, Popek EJ, Vogel H, Hicks J (2013). Pericardial effusion and cardiac tamponade in neonates: sudden unexpected death associated with total parenteral nutrition via central venous catheterization. Ann Clin Lab Sci.

[4] MacDonald MG, Rais-Bahrami K Rais-Bahrami K,  Ramasethu J  Ramasethu J (1983). Atlas of Procedures in Neonatology. https://www.google.co.in/books/edition/Atlas_of_Procedures_in_Neonatology/ZVNp9GvNb10C?hl=en&kptab=overview.

